# Efficacy of a novel patient-focused intervention aimed at increasing adherence to guideline-based preventive measures in asplenic patients: the PrePSS trial

**DOI:** 10.1007/s15010-023-02088-7

**Published:** 2023-09-01

**Authors:** Johannes Camp, Marianne Bayrhuber, Natascha Anka, Valerie Heine, Manuela Glattacker, Erik Farin-Glattacker, Siegbert Rieg

**Affiliations:** 1 Division of Infectious Diseases, Department of Medicine II, Faculty of Medicine, University of Freiburg, Freiburg, Germany; 2 Section of Health Care Research and Rehabilitation Research, Faculty of Medicine, University of Freiburg, Freiburg, Germany

**Keywords:** Asplenia, Post-splenectomy sepsis, Vaccination, Health action process approach

## Abstract

**Purpose:**

To determine whether a novel intervention improves the adherence to guideline-based preventive measures in asplenic patients at risk of post-splenectomy sepsis (PSS).

**Methods:**

We used a prospective controlled, two-armed historical control group design to compare a novel, health action process approach (HAPA)-based telephonic intervention involving both patients and their general practitioners to usual care. Eligible patients were identified in cooperation with the insurance provider AOK Baden-Wuerttemberg, Germany. Patients with anatomic asplenia (*n* = 106) were prospectively enrolled and compared to a historical control group (*n* = 113). Comparisons were done using a propensity-score-based overlap-weighting model. Adherence to preventive measures was quantified by the study-specific ‘Preventing PSS score’ (PrePSS score) which includes pneumococcal and meningococcal vaccination status, the availability of a stand-by antibiotic and a medical alert card.

**Results:**

At six months after the intervention, we estimated an effect of 3.96 (95% CI 3.68–4.24) points on the PrePSS score scale (range 0–10) with mean PrePSS scores of 3.73 and 7.70 in control and intervention group, respectively. Substantial improvement was seen in all subcategories of the PrePSS score with the highest absolute gains in the availability of stand-by antibiotics. We graded the degree of participation by the general practitioner (no contact, short contact, full intervention) and noted that the observed effect was only marginally influenced by the degree of physician participation.

**Conclusions:**

Patients who had received the intervention exhibited a significantly higher adherence to guideline-based preventive measures compared to the control group. These data suggest that widespread adoption of this pragmatic intervention may improve management of asplenic patients. Health insurance provider-initiated identification of at-risk patients combined with a patient-focused intervention may serve as a blueprint for a wide range of other preventive efforts leading to patient empowerment and ultimately to better adherence to standards of care.

**Supplementary Information:**

The online version contains supplementary material available at 10.1007/s15010-023-02088-7.

## Introduction

Asplenia or hyposplenic states are associated with a specific immunodeficiency rendering patients susceptible to invasive infections, the most severe manifestations referred to as post-splenectomy sepsis (PSS) [1–4]. Even if treated promptly and aggressively, morbidity and mortality remain high. However, most PSS episodes are preventable [1, 5]. An array of preventive measures, including vaccinations and some form of antibiotic prophylaxis, has been found to be effective in reducing mortality and has been adopted in many national and international guidelines [6, 7]. Adherence, however, is low with vaccination coverage among asplenic patients remaining unsatisfactory and patients oftentimes lacking adequate education regarding their medical condition [8]. This is especially unfortunate because well-educated patients have been shown to exhibit lower mortality rates from PSS [9].

Some researchers have evaluated and reported measures to strengthen guideline adherence such as spleen registries and dedicated outpatient services. Although some produced encouraging results, these implementations either depended heavily on local infrastructure or had shortcomings in the inter-sectorial communication (e. g. continuation of vaccinations post-hospitalization) [10, 11]. Therefore, widespread adoption seems unlikely. We hypothesized that adherence to recommended prevention measures can substantially be improved by a novel, health action process approach (HAPA)-informed intervention targeting both patients and their physicians. Several studies propose the HAPA theory as a theoretical framework for understanding health behavior in general [12, 13] and vaccination behavior in particular [14–16]. Here, we present data from the PrePSS study, a two-armed historical control group intervention study, which aimed to assess the impact of the developed intervention on guideline adherence in asplenic patients.

## Materials and methods

### Study design

We used a prospective controlled, two-armed historical control group design with baseline, post- and follow-up measurement. We decided against a randomized design because withholding critical information regarding post-splenectomy prevention measures even temporarily would expose patients not receiving the intervention to non-justifiable risks, thus violating ethical standards [9].

### Participants

Eligible were patients aged 18 years or older, with anatomic asplenia, a health insurance plan with AOK Baden-Wuerttemberg (Germany’s 5th largest health insurance that insures more than 4 million people) and at least conversational knowledge of German along with their respective physicians or other treating physician responsible for asplenia-related management. Patients who had received prior counseling at our institution were excluded. Eligible patients were identified by the AOK Baden-Wuerttemberg via a database search for OPS codes implying splenectomy (OPS code 5-413 splenectomy and sub-codes) and subsequently invited by mail to participate in the study. Patients who responded were asked to provide written informed consent and enrolled in their respective cohorts. Assignment to cohort was done based on prespecified time frames. Patients of the control group underwent splenectomy at least sixth month prior to study inclusion. Possible participants for the intervention group were identified on a biweekly basis by the health insurance provider and prospectively enrolled between February 2019 and January 2021. Upon enrollment patients were asked to provide their physician’s contact information and sign an exemption from confidentiality in order to allow for an exchange of information between the physician and the study center. Physicians whose patients consented to them being included in the study were subsequently contacted by mail and enrolled after having provided written informed consent. Both patients and physicians received a 30 € voucher upon study completion. The study was conducted as a single-center study at the University Medical Center Freiburg in cooperation with the insurance provider AOK Baden-Wuerttemberg, Germany.

### Intervention

An in-depth discussion of our intervention is detailed in the study protocol [17]. Briefly, our intervention consists of a patient-focused and a physician-focused intervention. Prior to the actual intervention patients and physicians were provided with tailored educational materials including a vaccination plan and a medical alert card for patients with asplenia. Due to the nature of our intervention blinding was not feasible.

### Patient-focused intervention

The patient-focused intervention was delivered as a telephonic, manual-based, individual intervention (T0) following the HAPA theory, combining an information-giving and motivational section and intervention components that promote motivation for initiation and planning of recommended infection prevention measures [12, 14, 15, 18].

In the information-focused and motivational section patients are made aware of the specific risks associated with asplenia (in particular PSS) and the recommended prevention measures, specifically vaccinations against pneumococci, meningococci, *H. influenza* and influenza virus, possession of a medical alert card and availability of stand-by antibiotic are introduced. The efficacy of the preventive measures is illustrated, and the presented information is framed by highlighting the patient’s personal relevance in order to promote risk perception, task self-efficacy and positive outcome expectancies. Afterward patients are encouraged to formulate individual prevention goals.

In a concluding planning section patients are asked to develop individual action plans to attain the self-set goals. Additionally, potential barriers (e.g., making appointments with the physician) are discussed and patients are encouraged to formulate coping plans to overcome these barriers.

Sixth months after the intervention patients in the intervention group were followed up via telephone call (T1), and the implementation of the recommended prevention measures was assessed using the PrePSS score. Where indicated, barriers to implementation were discussed and participants were assisted in managing difficulties. The follow-up consultation was not manual-based. Patients who discontinued the intervention or were lost to follow up prior to T1 were excluded from analyses because in these cases no data from T1 were available.

As per study design patients in the control group were identified more than six months after splenectomy, thus having received at least six months of standard of care.

### Physician-focused intervention

The physician-focused telephone intervention comprises evidence-based information concerning asplenia and the associated infections risks. Physicians are provided with both general information regarding recommended vaccinations in asplenic patients and tailored vaccination plans for the patient in question. Additionally, an introduction to the medical alert card is offered and necessity and feasibility of stand-by antibiotics are discussed. Although being primarily information-centered, the intervention includes motivational aspects, too, by highlighting the efficacy of preventive measures in averting PSS. Physicians who did not respond to the invitation letter and physicians of control group patients were contacted by telephone, and a short consultation was carried out in order to deliver the critical information regarding the patient at hand. In these cases, general information regarding asplenia was not included.

Manuals for both patient- and physician-focused interventions as well as a template for the medical alert card are provided in the supplement.

### Funding source

The study is funded by Innovationsausschuss of the Gemeinsamer Bundesausschuss, Wegelystraße 8, 10623 Berlin (grant number: 01VSF17049). The funding body was not involved in any aspect of the design of the study, collection of study data, in writing the manuscript or in the decision to submit this article for publication.

### Outcomes

Primary outcome was the adherence to preventive measures six months after the intervention compared to standard of care in the historical control group (assessed at least six months after splenectomy yielding similar time frames for preventive measures in both study arms). Adherence was measured by the study-specific PrePSS score, which assesses the following items: (a) receipt of guideline-conform sequential pneumococcal vaccination and (b) guideline-conform meningococcal vaccinations, (c) prescription and availability of stand-by antibiotics for emergency treatment and (d) handing out of and carrying a medical alert card. A total of 16 international experts in the care for asplenic patients were asked to rate the proposed guideline-derived items in terms of their importance in infection prevention. The scoring system employed in this study is derived from the median ratings of the nine experts who provided feedback. Additional details on the development of the PrePSS score have been described previously [17]. The PrePSS score was calculated based on the information gathered by the study physicians during both the patient- and the physician-focused intervention or during the assessment in the control group (Table S1). Because not at all physicians were able to receive the full intervention, we graded the level of physician participation (no contact, short contact, full intervention) and explored its association with the main outcome by comparing mean PrePSS scores.

Secondary outcomes included proximal HAPA-related variables such as patient’s risk perception, self-efficacy or action planning and more distal outcomes such as disease knowledge and health-related quality of life. Evaluation of these secondary outcomes will be reported elsewhere.

### Statistical analysis

Categorical variables are given as absolute and relative frequencies, continuous variables as means with standard deviation or medians with the first and third quartile. We adjusted for possible residual confounding due to missing randomization by using overlap weights, which is a propensity score weighting method [19]. As a sensitivity analysis we calculated inverse probability of treatment weights (IPTW) as well. We estimated the propensity score (PS) using a logistic regression model including all covariates listed in Table 1 as main effects. Overlap weights were then generated by assigning treated individuals the weight 1-PS, while those in the control group were assigned the weight PS. For the IPTW analysis weights were assigned as 1/PS and 1/(1-PS) for treated and untreated individuals, respectively.

**Table 1:** Baseline covariates before and after weighting using overlap weights

Variable	Intervention group (unweighted, *N* = 106)		Control group (unweighted, *N* = 113)		Intervention group (weighted, *N* = 102.8)		Control group (weighted, *N* = 109.3)	
	N/M (SD)	%	N/M (SD)	%	N/M (SD)	%	N/M (SD)	%
Age	58.3 (16.0)		58.1 (16.1)		58.6 (15.7)		58.6 (15.9)	
Sex								
Female	63	59.4	57	50.4	57.3	55.8	60.9	55.8
Male	43	40.6	56	49.6	45.5	44.2	48.3	44.2
Socio-economic status^1^	5.64 (1.74)		5.45 (1.75)		5.56 (1.74)		5.56 (1.75)	
Cause of splenectomy								
Trauma	17	16.0	25	22.1	18.7	18.2	19.9	18.2
Malignoma	48	45.3	46	40.7	44.5	43.3	47.3	43.3
Benign tumor	21	19.8	21	18.6	20.5	19.9	21.8	19.9
Hemato-oncological indication	9	8.5	9	8.0	7.9	7.7	8.4	7.7
Other	11	10.4	12	10.6	11.2	10.8	11.9	10.8
Subjective disease knowledge^2^	2.64 (0.95)		2.55 (0.91)		2.60 (0.93)		2.60 (0.91)	
Charlson comorbidity index^3^	2.84 (2.43)		2.78 (2.57)		2.83 (2.46)		2.83 (2.58)	
Immunosuppression (yes)	16	15.1	9	8.0	11.0	10.7	11.7	10.7

^1^MacArthur scale (1 = low social status, 10 = high social status)

^2^Subjective disease knowledge (1 = no knowledge about asplenia, 5 = high knowledge about asplenia)

^3^Charlson comorbidity index (severity of comorbidities 0 = none, 1–2 = mild, 3–4 = moderate, 5–33 = severe)

Our main reasons for using overlap weights were, firstly, that no observations are excluded from the outcome estimation in contrast to, e.g., propensity score matching. Secondly, overlap weights in contrast to other frequently used methods, e.g., propensity score matching, show less bias and seem more efficient [20].

The average treatment effect in the weighted samples was estimated using a generalized linear model, and confidence intervals were calculated using bootstrapping since the robust variance estimator is biased for propensity score weighting methods [21]. All estimated parameters are presented with their 95% confidence intervals. The whole analysis was conducted using R version 4.1.2.

### Ethical consideration

The study and intervention were approved by the Ethics Committee of the Albert-Ludwigs-University, Freiburg, Germany (vote no. 380/18). We followed the ethical standards set by the Helsinki Declaration of 1975, as revised in 2004. The PrePSS study is registered in the German Clinical Trials registry (DRKS00015238).

## Results

**Fig. 1:**
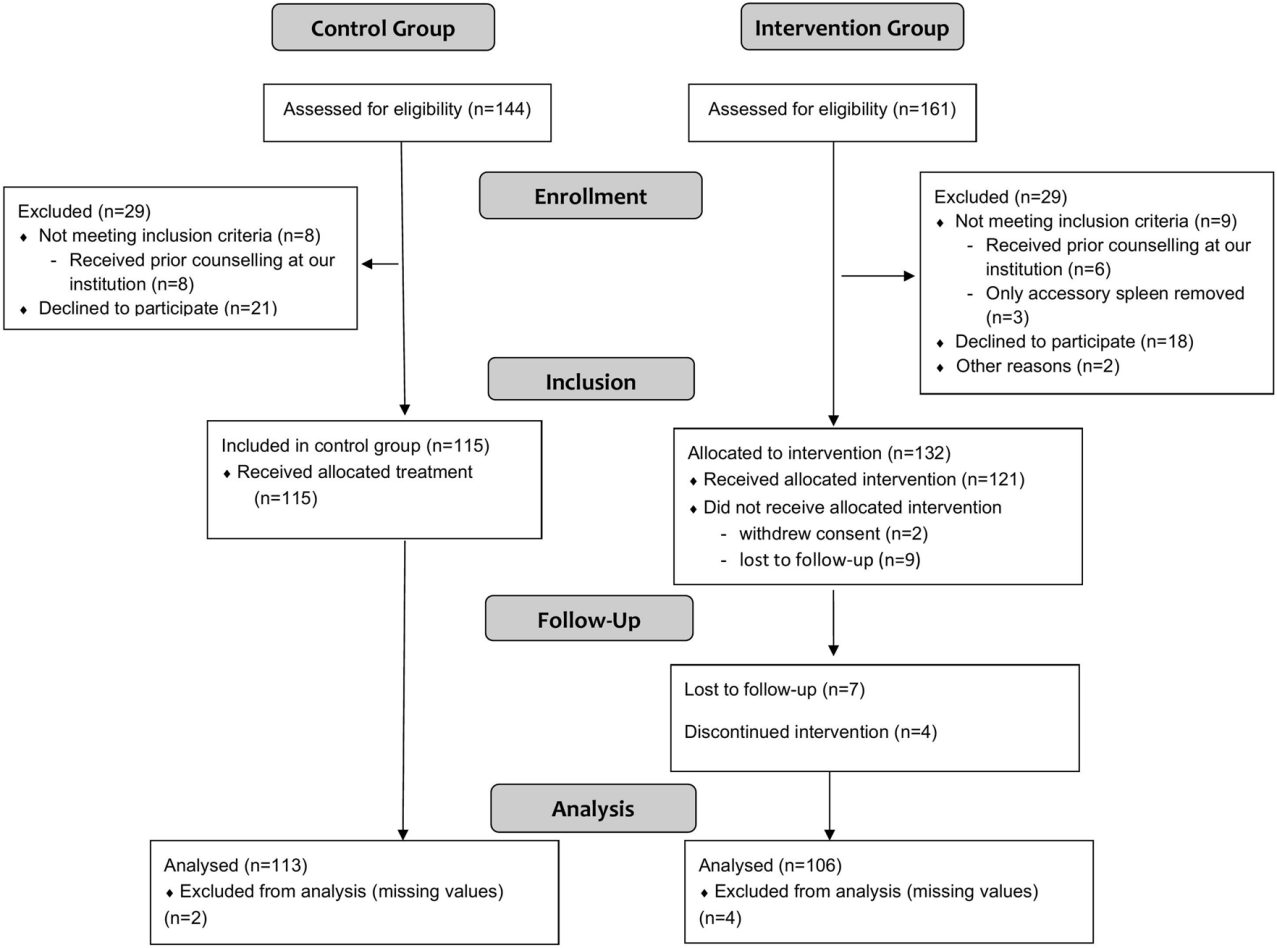
Flowchart of patient inclusion

A total of 247 patients were included in the study, 209 of which we were able to include in our analyses (Fig. 1). In total, 106 were included in the intervention group and 113 served as a historical control group. Although our study was not randomized due to ethical concerns, baseline characteristics were quite balanced even without weighting (Table 1). Main cause for splenectomy in both groups was an abdominal tumor (65.1% and 59.3%, respectively), of which the majority was malignant. Patients in the intervention group reported slightly higher rates of comorbidities and concurrent immunosuppression. The median time between splenectomy and study inclusion was 437 days (IQR 350–597) in the control group and 111 days (IQR 91–139) in the intervention group. After applying overlap weights, baseline covariates were exactly balanced and 102.8 and 109.3 patients were analyzed in the intervention and control group, respectively. Patients were 58.6 years old and a slightly higher prevalence of female gender was noted (55.8%). We recorded one PSS event in the control group and no events in the intervention group.

**Fig. 2:**
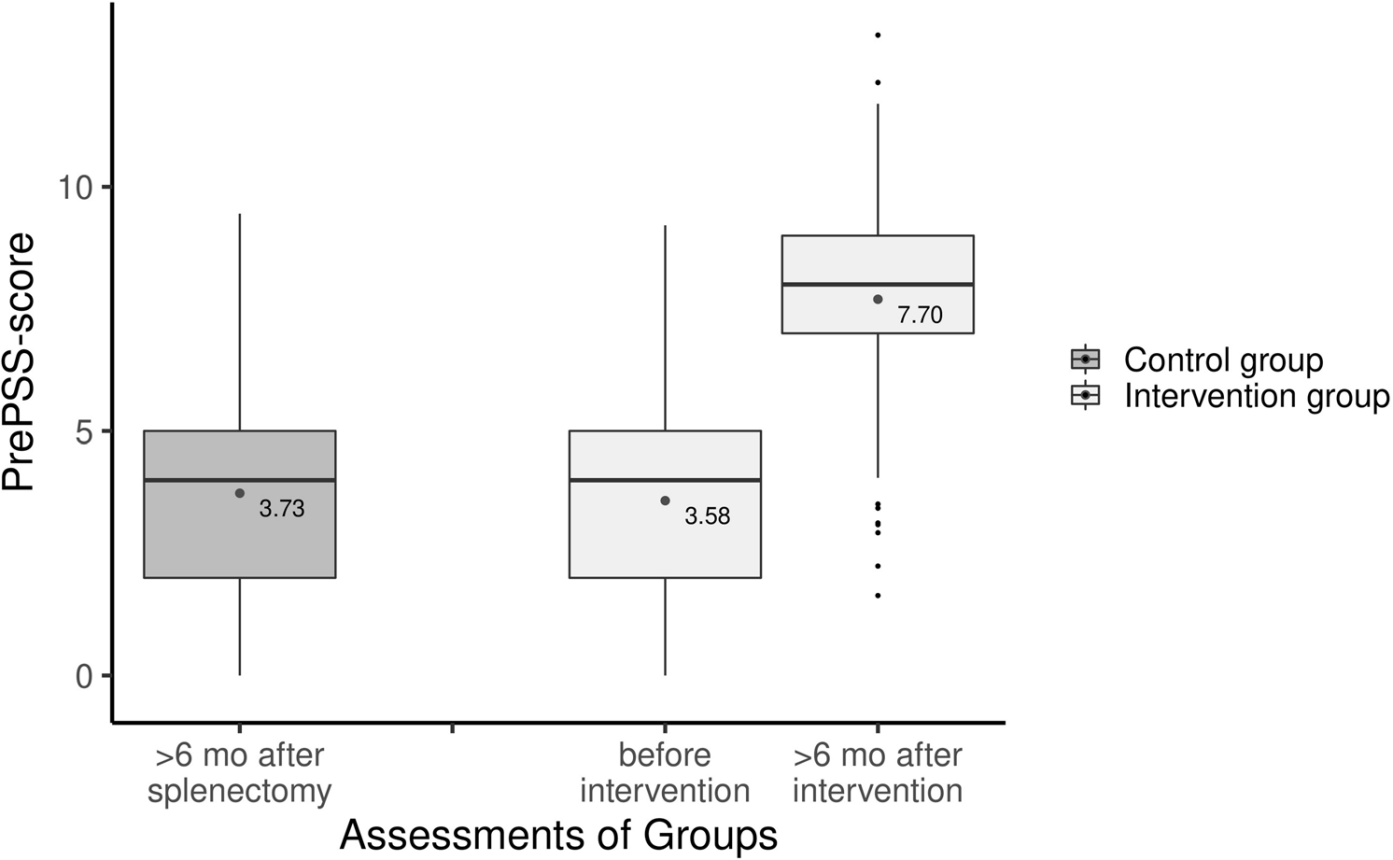
PrePSS score in the historical control group and the intervention group before (at T0) or > 6 months after the intervention (T1). Boxplots show median (black line) and interquartile range (box). Whiskers extend to ± 1.5 * interquartile range. Small black dots represent outliers; medium gray dots represent the mean PrePSS score as given in the annotation

At six months after the intervention, patients of the intervention group showed a mean increase of 3.96 (95% CI 3.68–4.24) points on the PrePSS scale [range 0–10], indicating a major improvement in guideline adherence compared to the historical control group (Fig. 2). Mean PrePSS scores were roughly doubled with 3.73 and 7.70 in control and intervention group, respectively. As a sensitivity analysis, we repeated the calculations using IPT weighting. Results were virtually unchanged with a mean increase of 4.02 (95% CI 3.77–4.26) points on the PrePSS scale.

**Fig. 3:**
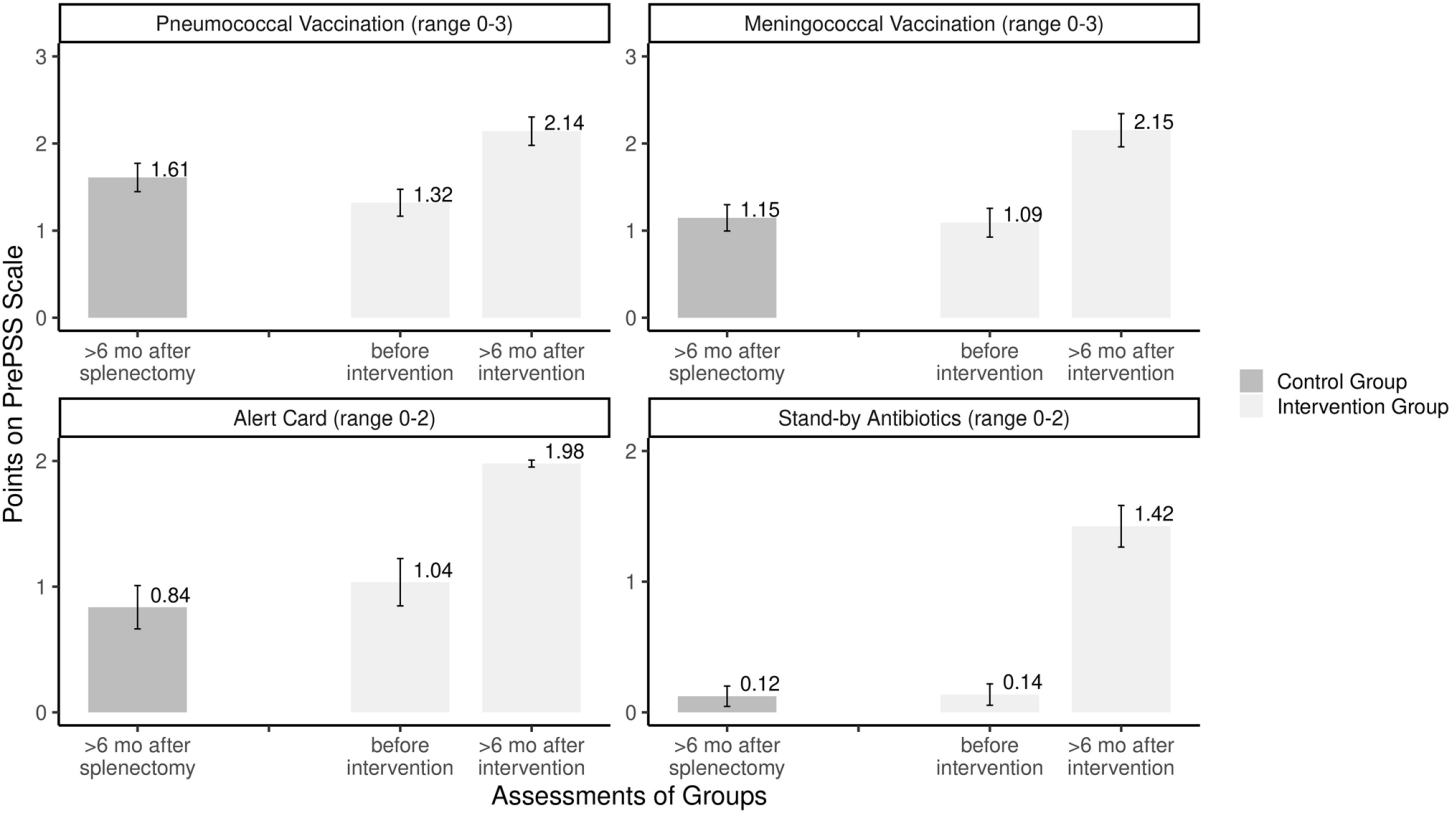
Mean points for different items of the PrePSS score in the historical control group and the intervention group before (T0) or > 6 months after the intervention (T1)

There were some marked differences between the individual items of the PrePSS score (Fig. 3 and Table 2). While most patients in the control group did receive at least one anti-pneumococcal vaccination, fewer than half of these patients were given a medical emergency card and fewer than 10% were in possession of a stand-by antibiotic. After the intervention, almost all patients (97.9%) carried their emergency card at all times and nearly two-thirds had a stand-by antibiotic readily available. An increase in complete meningococcal vaccinations was noted with 48.7% percent being fully vaccinated compared to 7.5% in the control group. Complete sequential vaccination against pneumococci did not see an equally noticeable gain, with only 42.2% of patients having completed this course after the intervention. However, this is almost double the rate of the control group and almost three quarter (73.0% vs. 41.7% in the control group) of the patients did in fact receive both anti-pneumococcal vaccines, albeit not in the recommended order. Overall, 21.4 patients in the intervention group achieved the full PrePSS score of 10 points, while no patient in the control group reached this level.

**Table 2:** Scores of individual PrePSS score items stratified by group

Variable	Control group (*n* = 109.3)		Intervention group T0 (*n* = 102.8)		Intervention group T1 (*n* = 102.8)	
	*N*	*P*	*N*	*P*	*N*	*P*
Guideline-conform sequential pneumococcal vaccination^1^						
3 Points	24.5	22.4%	14.2	13.8%	43.4	42.2%
2 Points	21.0	19.3%	11.6	11.3%	31.7	30.8%
1 Point	60.4	55.3%	69.9	68.0%	26.6	25.9%
0 Points	3.3	3.0%	7.1	6.9%	1.1	1.1%
Guideline-conform meningococcal vaccination^2^						
3 Points	8.2	7.5%	6.4	6.2%	50.1	48.7%
2 Points	19.6	17.9%	23.2	22.6%	26.4	25.7%
1 Point	61.5	56.2%	46.6	45.3%	18.1	17.6%
0 Points	20.0	18.3%	26.6	25.9%	8.2	7.9%
Handing-over and carrying a medical alert card						
2 Points	38.3	35.0%	50.1	48.8%	100.7	97.9%
1 Point	14.9	13.6%	6.2	6.0%	2.1	2.1%
0 Points	56.1	51.4%	46.5	45.2%	0.0	0.0%
Stand by-antibiotic prescribed and available (‘pill in the pocket’)						
2 Points	3.4	3.1%	3.1	3.0%	65.6	63.8%
1 Point	6.6	6.0%	7.8	7.6%	15.2	14.8%
0 Points	99.3	90.8%	91.9	89.4%	22.0	21.4%

^1^13-valent conjugate vaccine PCV-13 (Prevenar-13®) after ≥ 2 months followed by 23-valent polysaccharide vaccine PSV-23 (Pneumovax®)

^2^Tetravalent meningococcal conjugate vaccination Men-ACWY (Menveo®, Nimenrix®), two doses at least two months apart; meningococcal serotype B vaccine Men-B (Bexsero® [two doses] or Trumenba® [three doses])

Furthermore, we explored the association between the degree of physician participation in the intervention and the PrePSS score outcome. Many physicians did not receive the complete physician-focused intervention, primarily due to lack of time on the part of the physician. In these cases, a condensed version of the intervention focusing on the patient at hand was presented. Some physicians could not be reached at all. We graded the level of the received intervention (no contact, 13.8 patients; short contact, 64.0 patients; full intervention, 25.0 patients) and explored its relationship with the PrePSS score outcome (Fig. S1). Even patients, whose physician could not be reached at all, exhibited only marginally lower PrePSS-scores compared to the patients, where the physician received the full intervention, although a slight trend was noticeable.

## Discussion

Using a prospective controlled, two-armed historical control group design, we were able to demonstrate that our novel telephone-based HAPA intervention targeting both patient and physician led to considerably improved adherence to recommended preventive measures in asplenic patients. Educating and motivating the patient was identified as the crucial factor of the intervention since even patients, whose physician did not receive the intervention, scored only marginally lower than those, whose physician had received some form of intervention.

Integral part of all PSS prevention is the implementation of a vaccination plan. Due to different study designs and methodological approaches, investigators report a wide range of vaccine coverage in asplenic patients [5, 8, 22–24]. Most of the studies reporting high vaccine coverages report data from a “one-stop-shop” approach with clinicians administering vaccines during the hospital stay after splenectomy. This strategy, although effective initially, might prove challenging in the long term, because patients generally rely on their physician for booster shots and continuation of initiated vaccination schedules. Accordingly, studies with a more population-based approach reveal that vaccination coverage in asplenic patients in general is still unsatisfactory [8]. Where data regarding stand-by antibiotics are available, rates tend to be low, which is in good agreement with our findings [22, 25].

Several attempts have been made to improve guideline adherence. One popular approach is to establish an outpatient service in order to follow up asplenic patients and provide them with the necessary education and, if needed, vaccinations [10, 26]. Although effective and conceivably producing a sustained response, this approach is resource-intensive and by design regionally limited. Improvements in vaccination coverages have also been reported for implementations of local registries [11, 27]. However, these strategies depend on activities of local research groups; therefore, outreach is limited and sustainability often difficult to accomplish. Our intervention aimed to improve on some shortcomings of other approaches. Firstly, our intervention addresses the need for patient education. Although patients’ disease knowledge has long been recognized as crucial in disease prevention in general [28, 29] and specifically in prevention of PSS [9], surveys find that many patients did either not receive disease-specific education at all or have poor recollection of the delivered educational contents [30]. In our cohort, too, most patients stated that they had not been made aware of the long-term implications of splenectomy and were surprised by our initiative for the purpose of further education. We tried to address this issue by providing easy to understand information concerning asplenia in our telephonic intervention. Importantly, we integrated HAPA-informed elements, which were designed to involve the patients and achieve a sustained effect by treating the patients not as mere recipients of information but as actors in the process of their healthcare planning. It should be emphasized that our theory-based intervention takes into account both educational and volitional aspects to ensure that asplenic patients are empowered to apply prevention measures in the long term.

Secondly, our intervention is easily distributable, resource-saving when compared to face-to-face contacts and not confined to any specific setting in terms of both place and time. It can be understood as a telehealth preventive intervention, well applicable for example in underserved rural areas, and does not need dedicated structures but instead leverages existing ones, e. g. by involving the physician of the patient. By making our manual publicly available, we hope to open up a wide range of possibilities for implementation both in the inpatient and outpatient setting.

Thirdly, our intervention sought to bridge the intersectorial divide by involving the physicians in the prevention process. Intensifying the collaboration between clinicians and physicians was shown to be essential in order to improve the quality of care for asplenic patients [31]. However, we found that many physicians could not fully participate in the intervention as planned. In particular, with the added workload of the COVID-19 pandemic, most of them simply could not spare the time to participate in our study. Interestingly, though the level of asplenia-specific education, the physician received on our part was not heavily associated with PrePSS score outcomes. This further underlines the considerable potential of patient-focused interventions aiming for education and empowerment of patients with regard to their health or disease management. The role of the physician remains pivotal, however, since in Germany 85–90% of all vaccinations are carried out by the physician [32]. The physician’s contribution is especially essential in cases where patients are less well suited for our intervention due to, e.g., old age or comorbidities.

Finally, a unique feature of our design lies in the important role it assigns to the health insurance provider. Our approach includes an easy to perform and inexpensive screening procedure that enables health insurance companies to actively take part in delivering preventive strategies in a focused and patient-specific way. Leveraging the existing infrastructure of the health insurance provider not only facilitated inclusion of patients but also may prove pivotal in further implementation and widespread roll-out of our intervention. Given its aptitude for printed distribution, part of the intervention could conceivably be automated and sent out to both patients and their physicians upon screening and identification via OPS codes.

There are some limitations to our study. It is possible that our results are influenced by a responder bias, since first contact with patients was made via mail from the health insurance provider and we could only include those that responded to this letter. This might have led to an overrepresentation of patients, who were already motivated to obtain additional asplenia-related information. Our study was performed in cooperation with a singular health insurance provider. A potential barrier to widespread implementation could lie in the highly diverse landscape of the German health insurance system. While in theory all insurance companies should have access to the required data, the ability to implement the described intervention could conceivably vary depending on the type of insurance company (e.g., state funded vs. privately owned) and on the region in question. Additionally, due to the non-randomized design of our study, we cannot rule out confounding. Although we used a well-designed statistical model to account for this, unmeasured confounding might still be present. Due to the short follow-up time, we cannot speak to the long-term success of our intervention, yet we are currently performing a follow-up concerning primary and secondary outcomes three years after the intervention.

In conclusion, asplenic patients who had received a HAPA-based, telephonic intervention exhibited a significantly higher adherence to guideline-based preventive measures compared to a historical control group. Widespread adoption of this pragmatic intervention could improve patient care and ultimately lead to reduced morbidity and mortality from PSS. Moreover, our strategy of health insurance provider-initiated identification of at-risk patients followed by a patient-focused intervention may serve as a blueprint for a wide range of other preventive efforts leading to patient empowerment and ultimately to better adherence to standards of care.

### Supplementary Information

Below is the link to the electronic supplementary material.

Supplementary file1 (DOCX 1006 KB)

Supplementary file2 (DOCX 195 KB)

Supplementary file3 (PDF 641 KB)

Supplementary file4 (PDF 1859 KB)

## Data Availability

Publication of the dataset is not planned.
