# Supplementary material for: Efficacy of a novel patient-focused intervention aimed at increasing adherence to guideline-based preventive measures in asplenic patients: the PrePSS trial
Source: Infection. 2023 Sep 1;51(6):1787–95. doi: 10.1007/s15010-023-02088-7 (PMC10665246; doi:10.1007/s15010-023-02088-7)
Supplement: Supplementary file 1 — Supplementary file1 (DOCX 1006 KB) [file 15010_2023_2088_MOESM1_ESM.docx]

| **Manual**  **„Training for patients with asplenia and their physicians“**    **PrePPS Project** |
| --- |

Project lead:

Prof. Dr. Siegbert Rieg

Prof. Dr. Erik Farin-Glattacker

Dr. Manuela Glattacker

Scientists:

Dr. Marianne Bayrhuber

Natascha Anka

Dr. Johannes Camp


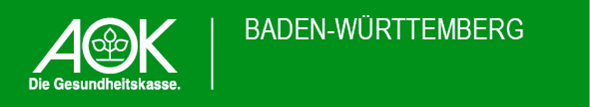

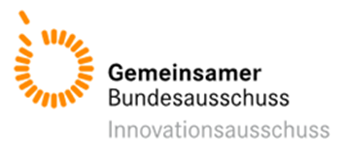

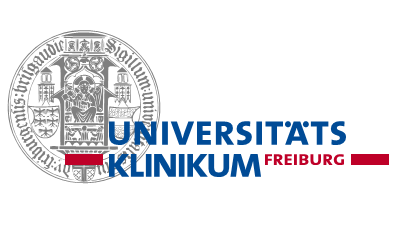
Version 1 – Updated 25.02.2019

*Aus Gründen der besseren Lesbarkeit wird auf die gleichzeitige Verwendung männlicher und weiblicher Sprachformen verzichtet. Das heißt, die Personenbezeichnungen gelten gleichermaßen für Frauen und Männer.*

| Table of contents |
| --- |

**1. Introduction**

1.1 Aims of the training

1.2 Target group

1.3 Theoretical Background

1.4 Summary of the intervention

1.5 Methods

1.6 Material

1.7 Trainer qualification

1.8 Organisation of the training

**2. Module 1: Telephone intervention for asplenic patients**

2.1 Learning goals of the module

2.2 Overview of the intervention

2.3 Schedule

2.3.1 Introduction

2.3.2 Education on guideline-based preventive measures

2.3.3 Motivation for implementation of preventive measures

2.3.4 Planning of preventive measures and barrier management

2.3.5 Conclusion

2.4 Material: Module 1

2.4.1 Patient education material

**3. Module 2: Telephone intervention for doctors treating patients with asplenia**

3.1 Learning goals of the module

3.2 Overview of the intervention

3.3 Schedule

3.3.1 Introduction

3.3.2 Education about risks and guideline-based infection prophylaxis

3.3.3 Conclusion

4 Literature

| Introduction |
| --- |

In Germany, approximately 8000 people undergo a splenectomy every year [1] for various indications including trauma (~20%), malignancy (solid tumours ~30%, hematologic neoplasia ~30%) and therapeutic splenectomy (~20%). It is estimated that one in 1000 German citizens in Germany have anatomic asplenia [2]. That number does not include patients with functional hyposplenia or asplenia, defined as conditions with partial or complete loss of function of the spleen. These conditions can arise in consequence of immunologic, hematooncologic or vascular pathologies as well as hemoglobinopathies. With an estimated prevalence of approximately 0.5%, the number of patients with functional hypo- and asplenia exceeds those with anatomical asplenia [3] by far. Anatomical and functional asplenia leads to a lifelong increased risk of severe invasive bacterial infections, mostly caused by pneumococci. New investigations report an incidence of 7-8 infections requiring hospitalisation per 100 patient years and an incidence of post-splenectomy sepsis (PSS) of one per 100 patient years. Compared to the general population the risk of sepsis-related hospital admission is 6-fold increased [4]. Even today, post-splenectomy sepsis has a hospital mortality of 30-50%. Patients surviving a severe course of the disease can face complications such as gangrene of fingers and toes up to a loss of limbs as well as potential consequential damage due to specific organ manifestations (meningitis, endocarditis). Quality of life and ability to work are often heavily impaired [2] after post-splenectomy sepsis. A recent multicentre prospective cohort study from Germany evaluated the vaccination status of patients being treated in intensive care units for post-splenectomy sepsis. Only 21% of patients had received a vaccination against pneumococci as recommended for asplenic patients within the past five years, only 6% had ever been vaccinated against meningococci and only 12% against *haemophilus influenzae* [1]. Only 12% of patients had received a seasonal influenza vaccine.

In the asplenia register study of the University Medical Centre Freiburg only 6% of patients being treated in a regular care setting by their primary care physician or specialist (not including patients who were treated at the aspenia outpatient clinic) were fully vaccinated according to current guidelines against pneumococci, meningococci and *haemophilus influenza* [5]. The data also show that the booster vaccination rates of the specific vaccines are even worse than the basic vaccination rates. Another finding from the study is that only minority of 47% of patients was prescribed a standby antibiotic.

To improve the care of patients with asplenia, the Division of Infectious Diseases of the University Medical Centre Freiburg (Prof. Dr. S. Rieg) and the Section of Health Care Research and Rehabilitation Research Sectsowie (SEVERA) (Prof. Dr. Erik Farin-Glattacker, Dr. Manuela Glattacker) developed a training for patients and their physicians as part of the PrePPS-project (PrePPS: Prevention of post-splenectomy sepsis in patients with asplenia).

The intervention aims to improve the implementation of and adherence to prevention measures substantially and sustainably. The expected outcome is a significant reduction of infections requiring hospitalisation and post-splenectomy sepsis. On the one hand, it should improve patient’s treatment result (reduction in morbidity and mortality) and improve their quality of life. On the other hand, the implementation of our planned intervention and preventive measures is also expected to improve the cost-effectiveness of the statutory health insurance since the cost for the training and preventive measures are relatively low compared to the cost of treatment and sequelae of post-splenectomy infections/sepsis.

The intervention is conducted via telephone by a trained study doctor of the Division of Infectious Diseases at the University Medical Centre Freiburg and is composed of a module for patients and a separate one for their physicians.

The **patient-focused intervention** takes up concepts about of health psychology, which have proven to be effective for the purpose of patient education [6]. Central to this approach is the inclusion of motivational and action-oriented competencies in training concepts that target objectives such as empowerment, shared decision-making or self-management (incl. improvement of adherence) instead of being solely restricted to delivering information [7].

The patient-focused intervention is based on the theoretical framework of the Health Action Process Approach (HAPA) [7], postulating a motivational phase in which intentions are formed concerning a particular health behaviour and a following volition phase in which the health bahaviour is planned, carried out and maintained. The taxonomy by Abraham & Michie provides the basis for the strategies employed here for behavioural modifications [8].

In the **physician-focused intervention**, emphasisis is placed on education about the consequences of a splenectomy and guideline-based preventive measures for asplenic patients. In accordance with the patient-focused intervention, motivational elements are included as well.

The manual is structured as follows: After an introduction (1), which provides information about the aims of the study, the target group, theoretical background, material, methods and organizational matters, both the patient-focused (2) and physician-focused (3) intervention are described in detail.

## 1.1 Aims of the training

Aims of the training:

1. Patients and their physicians are educated about guidline-based preventive measures for asplenia.
2. Patients and their physicians are motivated to adhere to those measures.
3. Patients acquire action-oriented competencies such as planning of preventive measures and barrier management.

Longterm goals of the training:

1. Reduction of severe infections requiring hospital admission and post-splenectomy sepsis through implementation of guideline-based preventive measures.
2. Improvement patient participation and compliance.
3. Improvement of a patient’s asplenia-specific self-management, health literacy and heath-related quality of life.

## 1.2 Target group

The training is directed at adults (18 years or older) with anatomic asplenia (partial or total surgical removal of the spleen) and their primary care physicians or specialists. The training is designed for both men and women. The time of the intervention is planned soon after the splenectomy to avoid complications such as severe invasive infections, in particular a post-splenectomy sepsis. Good knowledge of the German language in writing and speech is required for the training, which is conducted via telephone. To facilitate the transfer of aquired knowledge into everyday life patients are encouraged to discuss the intervention‘s content with their relatives.

## 1.3 Theoretical background of the intervention

The **patient-focused intervention** takes into account both motivational and educational aspects and is based on concept in health psychology known as the Health Action Process Approach (HAPA) [6]. The HAPA postulates a motivational phase with a focus on risk perception, outcome expectancy of a particular behaviour and self-efficacy, ultimately resulting in the formation of an intention (goal setting). Afterwards follows a volition phase, which is devided into three parts: a) Action Planning and Initiative, b) Action and Maintenance and c) Re-establishing or Disengagement after nonsuccess. Existing recources and situational barriers are important to consider for each phase. This intervention addressed all components of the HAPA. Beginning with the motivational phase the patient’s risk perception, outcome expectancy and self-efficacy are being strengthened by educating them about their increased risk of servere infections and feasable preventive measures. After setting of a goal follows the volition phase with planning of preventive measures and maintenance of the desired behavior as well as the management of possible obstacles. Strategies aiming at behavioural modifications are based on the taxonomy by Michie (2013). Michie et al. [8] described 93 techniques in sixteen categories useful for complex interventions. In the intervention on hand, we considered the following categories:

Goals and Planning: Preventive measures,

Social support: Relatives and physicians,

Shaping knowledge: Function of the spleen, emergencies, preventive measures, efficacy of the measures,

Salience of consequences: Education about the risk of severe infections in asplenic patients

Promts/cues and Associations: Calendar entry: vaccination dates, expiration date of stand-by antibiotic

The **physician-centered intervention** focuses on conveying information. Participating physicians are educated about the increased risk of severe infections in aplenic patients and current guideline-based preventive measures, especially vaccination recommendations. In accordance to the patient-focused intervention, the goal is to increase risk perception and outcome expectancy and consequently the motivation to implement recommended preventive measures.

**1.4 Structure of the intervention**

The intervention is comprised of a module for patients and a module for physicians and is conducted via telephone by a study doctor. Content of the intervention are education and strategies to improve the implementation of recommended preventive measure in asplenic patients.

| **Module** | **Duration** | **Topic** |
| --- | --- | --- |
| Module 1 | 20 minutes | Patient-focused module |
| Module 2 | 10 minutes | Physician-focused module |

### Module 1: Patient-focused module

The patient-focused module provides education about the guideline-based recommendations for preventive measures and aims to motivate and empower patients to implement those measures and manage possible barriers. Prior to the intervention patients are sent information material and an action plan for the particular preventive measures to improve the transfer of acquired knowledge into daily life. A follow up takes place six months later via telephone to discuss the implementation of preventive measures and possible barriers and – if required – another attempt at motivating patients to implement the recommendations. The telephone contacts are structured, guideline-based and results of the call documented.

**Module 2: Physician-focused module**

The physician-focused module aims to equip physicians with relevant and evidence-based information about the consequences of asplenia (risk of severe infections and associated high morbidity and mortality) and guideline-based recommendations for preventive measures. Contents and structure of the training concept were developed based on the emergency medical card (5^th^ edition) of the German Society of Infectious Disease (DGI) and German Sepsis Society for Patients with Asplenia (available here: www.asplenie-net.org).

In preparation for the telephone intervention, participating physicians recieve concise information material about asplenia and guideline-based preventive measures. Six months after the intervention, another telephone interview takes place to follow up on the implementation of preventive measures taken by the patients and the subsequent evaluation of those by their physician. The telephone contacts are structured and carried out according to guidelines analogue to their respective patient interviews.

## 1.5 Didactic methods

**Patient-focused training**

To meet the objectives of the training, various didactic methods were applied. Patients receive education and are included by being encouraged to ask questions and react to questions posed by the study doctor. Activating methods with an interactive character are used alongside in the training. Together with a study doctor, patients work out goals to plan and implement preventive measures and management of possible barriers. Participants receive material designed to help train and deepen their understanding.

In addition, the information material is intended to assist patients discussing the contents of the training with their relatives and thereby facilitate transfer into daily life.

**Physician-focused training**

The physician-focused training is designed in a way that study doctors give advisory information to the participating physicians. Emphasis is put on the offer to support the implementation of (new) preventive measures without restricting physicians in their freedom of decision or giving them binding instructions.

During the training physicians can ask questions or voice concerns about the feasibility of implementing particular preventive measures. Accompanying the training physicians receive information material and the medical emergency card as distributed to patients.

## 1.6 Materials

Prior to the telefone intervention, patients are sent educational material via postal service providing information about the spleen’s function, recommended preventive measures for patients with asplenia and how to behave in case of an emergency. In addition to the educational material, patients recieve a medical emergency card and an action plan for the telefone interview (see chapter 2.4.2).

Physicians also recieve written educational material about asplenia and appropriate preventive measure. It includes in particular the recommended vaccination schedule with a detailed plan on the sequence of vaccinations against pneumococci, meningococci and heamophilus influenzae. The information material includes a medical emergency card as distributed to patients.

## 1.7 Qualification of the trainer

The training should be performed by a doctor who is sufficiently schooled about the intervention and has experience treating and advising asplenic patients. It is important to respond individually to the participants and to deal constructively with possible disruptions.

Study doctors should avoid using foreign words and medical terminology while training patients and instead rephrase them into everyday language (chapter 4).

## 1.8 Organisation of the training

The training is conducted via telephone, which allows for inclusion of patients and physicians living further away. Each patient and physician receives a separate training by a study doctor, therefore obtaining the opportunity to integrate the training into everyday life.

| Module 1: Telephone intervention for asplenic patients |
| --- |

**Duration: ca. 20 minutes**

## 2.1 Goals of the module

The patient-focused intervention aims to improve the implementation of preventive measures and self-management of patients through their empowerment (defined here as the ability to make informed decisions regarding the particular measures). Motivational strategies and action-oriented competencies are conveyed alongside the information given throughout the training. Patients should be educated about the risk of severe infections in asplenia and learn about effective preventive measures. Furthermore, they should be motivated and enabled to carry out recommended preventive measures.

| **Topic** | **Guideline-based preventive measures for asplenic patients** |
| --- | --- |
|  |  |
| **Lead** | Study doctor |
| **Room** | Telephone contact |
| **Material** | Worksheet A1, Patient information of the Division of Infectious Diseases of the University Medical Centre Freiburg, Medical emergency card |
| **Goals** | Z.1 Relationship building  Z.2 Information about guideline-based recommendations for preventive measures  Z.3 Motivation to implement preventive measures: Improve risk perception, self-efficacy and outcome expectancy  Z.4 Planning of preventive measures and barrier management |

## 2.2 Overview of module 1

An overview of the patient-focused module 1 is shown in the following table. Outlined are the contents of each section, corresponding goals as well as the approximate time for the section and required materials.

Table 1

| **Goals** | | **Content** | **Duration** | **Materials** |
| --- | --- | --- | --- | --- |
| Z. 1 | Relation building | Introduction | ca. 2 min. | - |
| Z. 2 | Knowledge aquisition | Education about guideline-based preventive measures | ca. 4 min. | Patient information Worksheet A1, Medical emergency card |
| Z. 3 | Motivation | Education about the efficacy of preventive measures, emergencies | ca. 6 min. | Worksheet A2 |
| Z. 4 | Planning and barrier management | Individualised planning of preventive measures | ca. 6 min. | Worksheets A3 und A4 |
|  | - | Conclusion | ca. 2 min. | Refer to evaluation sheet |

**2.3 Schedule**

**2.3.1 Introduction**

*Goal 1: Relation building between doctor and patient*

The study doctor introduces himself by name, profession and function. He provides a short overview of the goals of the training: Providing information about easy to implement preventive measures for asplenic patients and their physicians. The aim is to avoid severe infections, which can progress to sepsis.

The study doctor encourages patients to ask **questions** in case of uncertainty about any topic and to voice **opinions or concerns**. Afterwards he expresses his gratitude for the patient’s participation in the study.

**Introduction** by name, profession and function

 “You recently underwent surgery removing your spleen and today we will discuss what can be done to prevent certain infections which are more likely to develop in this condition.The aim is to avoid serious infections that can lead to septicemia. I will introduce you to preventive measures and talk to you about what to do in an emergency.”

“If you have any **questions**, comments or concerns please feel free to interrupt me at any time.”

**2.3.2 Education about guideline-based recommendated preventive measures for asplenic patients**

*Goal 2. Knowledge aquisition*

After the introduction, the study doctor informs the patient about the function of the spleen and guideline-based preventive measures to protect against serious infections. He then provides information about recommended vaccinations against pneumococci, meningococci and influenza, as well as the so-called “stand-by antibiotic” and the medical emergency card.

„The **spleen** is important for the immune system and has both a filter function and a defense function. The spleen filters old blood components from the blood and is involved in the defense against pathogens. If the spleen is removed, the risk of serious infections, secondary diseases and complications is increased.

However, effective and simple **preventive measures** exist. The most important measure to protect against severe infectious is vaccination. Vaccinations against pneumococci, meningococci and the anual flu vaccine are recoomended.

A so-called „**emergency antibiotic**“ is also recommended, i.e. an antibiotic which is carried with you on a daily basis. The location of the antibiotic is known and accessible at all times.

Furthermore, it is recommended to carry a **medical emergency card** with you at all times, so that in an emergency treating physicians are informed about the asplenia and the consequences for further treatment.

You can find these preventive measure on your **Worksheet 1**.“

„Do you have any **questions**?“

**2.3.3 Motivation to implement preventive measures**

*Goal 3: Increase risk perception, self-efficacy and outcome expectancy*

In order to motivate patients to implement recommended preventive measures, the study doctor explains the efficacy of the measures and risk situations and refers to **Worksheet 2**.

„The **effectiveness** of the preventive measures has been scientifically proven. If patients without a spleen are well informed about their risks, septicemia occured much less frequently after the removal of the spleen. Over a 17-year period, onnly 15 ouf of 1000 patients who received education about their condition developed septicemia, whereas 164 out of 1000 patients without such education developed septicemia.

This can also be found on **Worksheet 2**.“ Dies finden Sie auch auf Arbeitsblatt 2.“

„Do you have any **questions**?“

“In patients without a spleen, severe infections can often develop abruptly, sometimes within a few hours. Therefore, every patient should know the **warning symptoms of blood poisoning**. These include:

- Fever > 38ºC or chills, feeling severly ill
- Symptoms of a severe flu infection
- Acute cofusion, drowsiness, headache
- Severe abdominal pain, nauea, diarrhea
- Abnormally rapid hearbeat and dizziness

If you notice these symptoms, you should immediately contact your family physician or go to the nearest hospital emergency room. If this is not possible, you should **start taking emergency antibiotics** without delay. In any case, a doctor must be consulted as soon as possible for further clarification of the condition.

Crucial is that there is a "strong feeling of illness", which is absent, for example, in a normal flu-like infection. If you feel severely ill and **unusually bad,** it is important to act quickly, i.e. visit a doctor or take emergency antibiotics. If you only have a common cold, it is not necessary to go to the emergency room.

„Do you have any **questions**?“

**Cave**:

**Cave**: At this point, the risk of serious infections should be objectively pointed out without invoking fears. Patients' expectation of self-efficacy to avoid infections through preventive measures should be strengthened.

**Objective: Implementation of preventive measures**

The study doctor refers the patient to **Worksheet 2** and repeats the recommended preventive measures (vaccinations, emergency medical card, stand-by antibiotic). He then proceeds to ask which of the measures the patient would like to implement.

“We talked about the recommended preventive measures for asplenic patients, i.e. vaccinations, the medical emergency card and the emergency antibiotics. Can you imagine **implementing these measures in your everyday life**? Or have you already implemented some of them?

On **Worksheet 2** below, you will find the precautionary measures mentioned. Which of these would you like to implement? Please tick your **goals**.”

**2.3.4 Individual planning of preventive measures**

*Goal 4: Planning and barrier management*

The study doctor plans the preventive measures together with the patient, asks about possible barriers and works out how to deal with them. To this end, questions are asked as listed on the worksheet under each measure. The same questions can be found on the patient’s **Worksheet 3**. After the joint planning of the preventive measures, the patient writes down the agreed procedure in the respective field on the worksheet. Feasable solutions can be found in the fields of the individual preventive measures further down in this section.

Vaccinations, emergency antibiotics and the medical emergency card are discussed in this order. If measures are not desired or have already been taken, the doctor moves on to the next point.

Useful strategies to deal with barriers are summarized table 2.

„You would like to implement the following preventive *measures (please tick the appropriate selection)*:

- **V**accinations against:
  - Pneumococci
  - Meningococci
  - *Haemophilus influenzae* type b
  - Flu (annually)
- **M**edical emergency card
- **A**ntibiotic

We know how difficult it is to get the flu shot every year. It has been shown that it is easier to implement preventive measures if they are well planned. Therefore, I would like to **plan this with you** and talk about **possible obstacles in everyday life**. You can enter our results on **Worksheet 3**.“

**Vaccinations** Already done Not requested


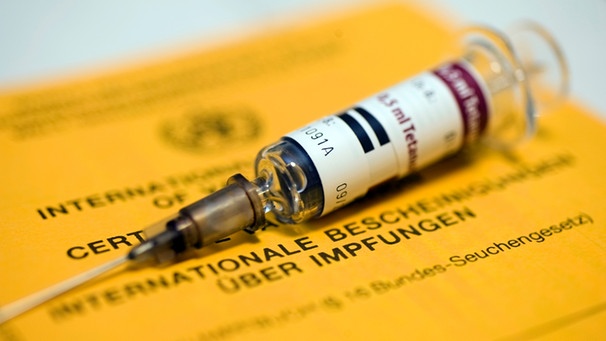
 **Agreed procedure**:

| - How can you plan your vaccination appointments?  - What can prevent you from going to the appointment?  - How can you handle that? | 1. Plan appointments 2. Go to the primary care physician to get the annual flu vaccine in October/November and ask the doctor to check if you need to renew any other vaccinations? |
| --- | --- |

**Medical emergency card** Already done Not requested

 **Agreed procedure:**

| - Where you keep your medical emergency card?  - In which situations could you forget to carry it with you?  - How can you prevent that?  - Is all your necessary information filled out in the card? | a*) Wallet*  *b) Husband/Wife carries a copy*  *c) Relatives can inform a doctor in case of emergency* |
| --- | --- |


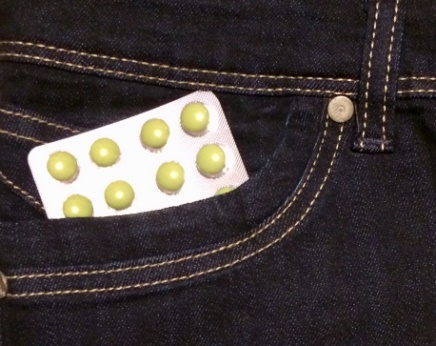
**Antibiotics** Already done Not requested

**Agreed procedure:**

| - Where you keep your antibiotics?  - In which situations might you not have it with you?  - How can you prevent that? - How do you ensure renewal once the prescription has expired? | *a) Wallet*  *b) Husband/Wife carries part of the prescription*  *c) Note the expiration date in a calender* |
| --- | --- |

An overview of possible concerns patients might express regarding the specific preventive measures is summarised in table 2. The study doctor works with the patient to work out how to deal with these barriers. If a patient excludes a measure for himself, the doctor accepts this decision.

Table 2: Dealing with barriers

| **Concerns about...** | **Management** |
| --- | --- |
| **Vaccination**  Occurence of severe adverse events, lack of efficacy, short life expectancy(i.e. tumor) | Benefit outweighs risk, Information about vaccinations The Standing Committee on Vaccination (STIKO) of the Robert Koch Institute |
| **Stand-by antibiotic**  Occurence of severe side effects, spread of resistant germs if used frequently, infection could be of viral origin and an antibiotic possibly harmful | Benefit by far outweighing risk, doctor involved in therapy, not a longterm medication |
| **Medical emergency card**  In the event of a serious illness, to convey to the doctor treating you that you have an immune deficiency (missing spleen) and need appropriate treatment | Include relatives, refer to asplenia outpatient clinic at the University Medical Centre Freiburg (possibly with relatives if required) |

**2.3.5 Conclusion**

In the end, the study doctor summarises which preventive measures the patients agreed to implement, explains the further proceedings and thanks the patient for the conversation.

Short **summary** of planned preventive measures:

Refere to further proceeding (next telephone interview, T1 questionnaire, evaluation sheet).

„Is there anything you did not fully understand or do you have any open **questions**?“

Say goodbye the patient and express your **gratitude** for the talk

**2.4 Materialien: Modul 1**


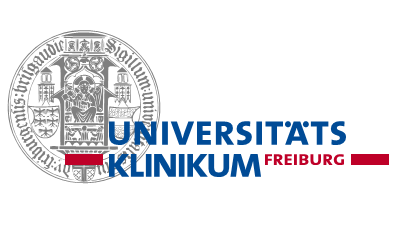
**2.4.1 Patient education material**

| Education material for patients |
| --- |

**Worksheet A1: Recommended prevention strategies for patients without a spleen
VMA (Vaccinations, Medical emergency card, Antibiotics)**

- **Vaccinations**

Immunizations are the most important preventative measure against severe infections. Currently recommended are pneumococcal and meningococcal vaccines, the *Haemophilus influenza* vaccine and annual influenza vaccination.


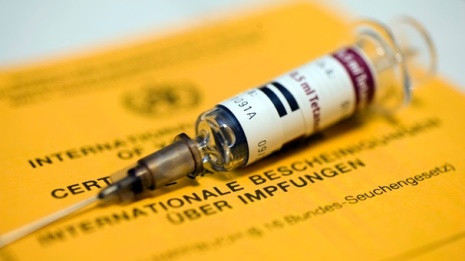


Bild: picture-alliance/dpa

- **Medical Emergency Card**

Carrying a medical emergency card at all times is also recommended. In an emergency, it informs doctors about the asplenia and ensuing consequences for treatment.

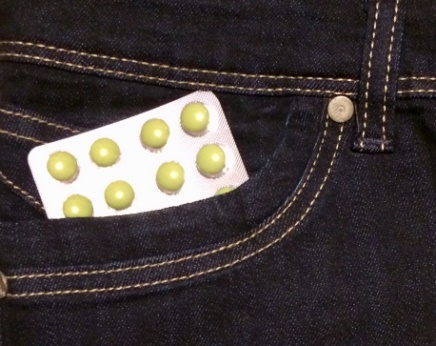


- **Stand-by Antibiotic**

Further recommended is the prescription of a “stand-by antibiotic”, an antibiotic readily available at all times after the prescription has been redeemed.

**Worksheet A2: Efficacy of preventive measures/Goals**

Occurrence of **septicemia** within a 17-year period in patients without a spleen


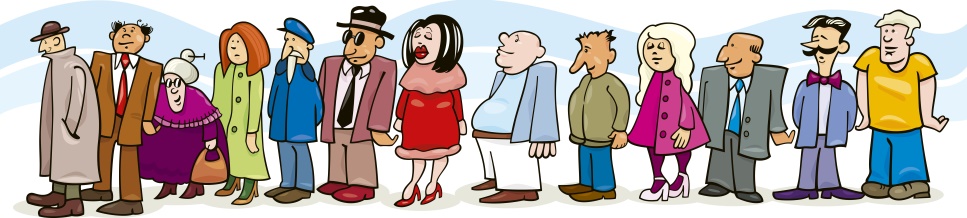


**Without** risk education a bloodstream infection occurs in 164 of 1000 patients

1000

600

400

800

200

**With** risk education a bloodstream infection occurs in only 15 of 1000 patients

Quelle: El-Alfy MS, El-Sayed MH. Overwhelming postsplenectomy infection: is quality of patient knowledge enough for prevention? Hematol J 2004;5(1):77-80.

**My goals: I want to implement the following precautions…**

Please check the boxes below that apply to you.

Pneumococcal and meningococcal vaccination, annual influenza vaccination

Carrying the medical emergency card

Carrying an antibiotic

**Worksheet A3: Planning of preventive measures**

**VMA (Vaccinations, Medical emergency card, Antibiotics)**

**Vaccinations** Already done Not requested


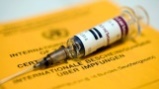


How do I plan my vaccination appointments? What could stop me from going to the appointment? How can I handle that situation?

**Agreed procedure**:

________________________________________________________________________________________________________________________________________________________________________________________________________________________________________________________________________________________________________

**Medical emergency card** Already done Not requested

Where do I keep my medical emergency card? In which situations might I forget to bring my card? How can I prevent that from happening?

**Agreed procedure:**

________________________________________________________________________________________________________________________________________________________________________________________________________________________________________________________________________________________________________

**Antibiotic** Already done Not requested


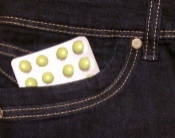


Where do I keep my stand-by antibiotic? Are there situations in which I might not have access to the medication? How can I prevent that from happening? How do I ensure renewal of my prescription after the expiration date?

**Agreed procedure:**

________________________________________________________________________________________________________________________________________________________________________________________________________________________________________________________________________________________________________

**Worksheet A4: Vaccination schedule for patients without a spleen**

|  | **Basic immunization** | | **Booster vaccines** |
| --- | --- | --- | --- |
|  | 1. **Vaccination** | 1. **Vaccination** |  |
| **Pneumococci***  (PCV-13, PSV-23) | Vaccination  (PCV-13) | 2 months later  (PSV-23) | 5-6 years later  (PSV-23) |
|  | If you already received a dose of PSV-23, the immunization with PCV-13 should be followed earliest at 12 months after the vaccination with PSV-23. | | |
| **Meningococci***  (Men-ACWY **or** Men-B) | Vaccination  (Men-ACWY) | 2 months later  (Men-ACWY) |  |
|  | Vaccination  (Men-B) | 2 months later  (Men-B) |  |
| ***Haemophilus influenzae* type b** | Vaccination  (Hib conjugate vaccine) | None | - |
| **Flu (Influenza)** | Influenza vaccine | - | Annual |

Status 03/2019

Please ask your doctor to document your vaccination dates in the medical emergency card.

*** Pneumococci, Menigococci:** There are two different vaccination preparations used.

| Module 2: Telephone intervention for doctors treating patients with asplenia |
| --- |

## 3.1 Learning goals of the module

The physician-focused module has the following learning objectives: The attending physicians should acquire knowledge about the health consequences of the spleen removal (risk of serious infections and the associated high morbidity and mortality) and about (new) preventive measures or update and expand existing knowledge.

This is intended to enable providers to make treatment-relevant decisions based on current evidence and to motivate them to implement recommended preventive measures.

| **Topic** | **Guideline-based preventive measures in asplenia** |
| --- | --- |
|  |  |
| **Lead** | Study doctor |
| **Place** | Telephone contact |
| **Material** | Information for attending doctors (Division of Infectious Diseases at the University Medical Centre Freiburg), Medical emergency card |
| **Goals** | Z.1 Relationship building between study doctor and medical provider  Z.2 Acquisition and update on the risk of infection in asplenia and guideline-based recommendations for infection prophylaxis  Z.3 Motivation to implement the preventive measures by increasing risk perception and outcome expentancy |

**3.2 Overview of the intervention**

An overview of the physician-focused invervention is given in the following table showing the contents of each section, associated goals, approximate duration and required materials.

| **Goals** | | **Content** | **Duration** | **Materials** |
| --- | --- | --- | --- | --- |
| Z. 1 | Relationship building | Introduction | ca. 3 min. | - |
| Z. 2  Z.3 | Acquisition and update of knowledge  Motivation | Education about risks and guidline-based recommendation for infection prophylaxis | ca. 6 min. | Medical emergency card |
|  | - | Conclusion | ca. 1 min. | Reference to homepage |

**3.3 Schedule**

## 3.3.1 Introduction

*Goal 1: Relationship building between study doctor and medical provider*

The study doctor introduces himself by **name, profession and position** and briefly explains the **aims** of the training: The comprehensive education of patients with asplenia and the provision of information on recommended (updated) preventive measures for their physicians.

**Cave**: At this point, the offer to provide information to the treating doctor should be emphasized as **support** in the implementation of preventive measures.

The study doctor then thanks the provider for participating in the study and points out questions can be asked at any time.

**Introduction** by name, profession and position

„As part of pur study, patients with asplenia are extensively trained about the risk of severe infections and necessary preventive measures. As you probably already know, the **aim** of this training is to **improve the implementation of appropriate measures for infection prophylaxis**.

Since we expect your patient to visit you in the near future, we **offer to give you a brief overview about current recommendations** grounded on evidence-based guidelines from national and international professional societies as well as relevant study results.

For one there are new vaccines and vaccination recommendations that have made vaccination plans more complex. We would also like to give you a brief introduction to the updated medical emergency card (5th edition) for patients with asplenia, which was developed by the German Society for Infectious Diseases and the German Sepsis Society, among others."

**Thank** the physician for participating

„If you have any question, please feel free to interrupt me any time.“

**3.3.2 Risikoaufklärung und Aufklärung über leitliniengerechte**

## 3.3.2 Education about risks and guideline-based recommendations on infection prophylaxis

*Goal 2: Knowledge acquisition and update
Goal 3: Motivation to implement the preventive measures by increasing risk perception and outcome expentancy*

After the introduction, the study doctors informs the participating physician about the health **consequences of the splenectomy** and about the existence of effective measures for infection prophylaxis.

**Education about the risk of infection after splenectomy:**

- „Asplenic patients have a **high lifetime risk** of developing severe invasive infections (post-splenectomy sepsis, PSS), most frequently caused by **pneumococci**, less often by **meningococci** and **H. influenzae**. Even today PSS still has a hospital mortality of 30-50% [2].
- The risk of infection is at its highest 2-3 years post splenectomy but remains lifelong and increases with underlying disease, thalassemia, medical immunosuppression or current chemotherapy [1].
- …

The study doctor points out how important it is that **patients are fully informed about the risks and preventive measures for asplenia** and have a **medical emergency card** that they carry with them at all times. If they have not already been introduced to it elsewhere, patients will receive such an emergency card as part of the study.

The study doctor then briefly explains the **structure and purpose of the medical emergency card.**

**Relevance** of the medical emergency card

„If they haven’t already done so, patients receive such a medical emergency card **as part of the study**.”

**Structure and purpose of the medical emergency card**

„The medical emergency card…

- …informs doctors in an **emergency** about the increased risk of sepsis
- …gives and overview about the most important **preventive measures** (pathogens and indicated vaccines with a vaccination schedule, indicated antibiotics)
- …offers the possibility to document vaccinations and vaccination dates as well as the stand-by antibiotic.“

**Medical emergency card**: „The German Society for Infections Disseases and developed such a medical emergency card which you have received together with all the other information material in the mail. On it, you can find all the measures for infection preventions, which I will talk about in detail during the course of this call“.

.

The study doctor points out the **existance and relevance of new vaccinations** and vaccination recommendations for infection prevention.

- „Vaccinations are the most important measure for infection prevention after splenectomy. Vaccines are available against the three most important pathogens causing sepsis: **pneumococci, meningococci and *Haemophilus influenzae***.
- In principle, conjugate vaccines are preferable, with the serotype spectrum of pneumococci being broadened with a booster vaccination of a pneumococcal polysaccharide vaccine.
- The best vaccine response is archieved two weeks prior to the elective splenectomy (otherwise 10-14 days post-splenectomy).
- In addition, the **annual flu vaccine** should be given because a flu infection predisposes to secondary bacterial infections, especially with pneumococci.”

The study doctor explains the vaccination schedule based on the vaccination plan printed on the medical emergency card.

**Vaccination schedule Updated 25.02.2019**


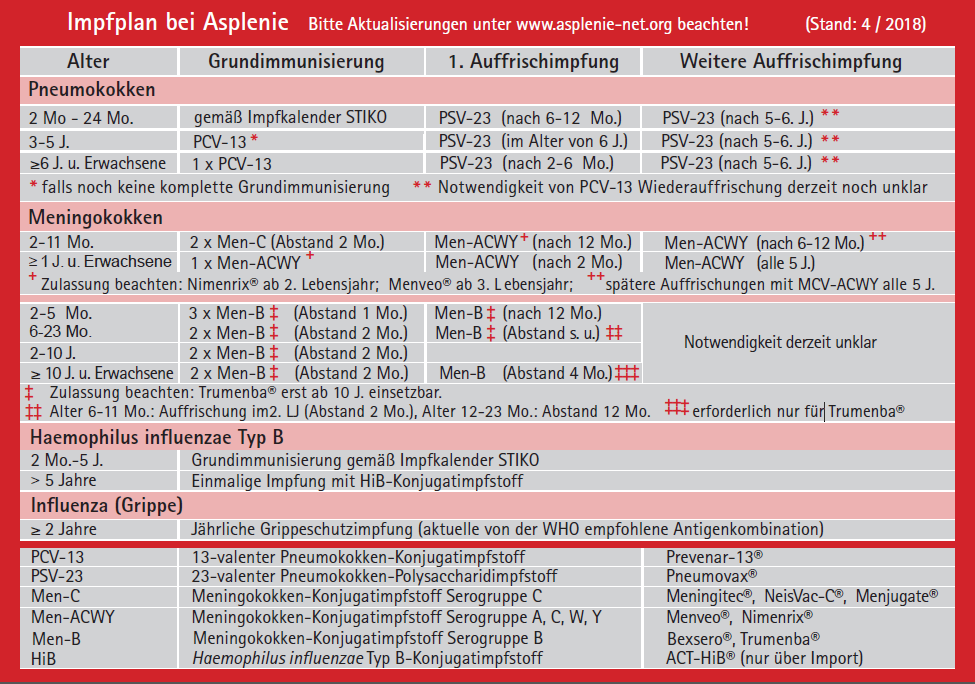
 **Cave: Current recommendations must be verified before using this scheme.**

The study doctor refers to the **back of the medical emergency card** and advises the provider to use this spreadsheet template to document the patient's individual vaccination dates (taking into account the specified vaccination schedule).

„On the **back** of the medical emergency card you can find a table in which a patient’s vaccination dates can be entered.

This approach has proven to be especially useful to improve compliance as the vaccine schedule is difficult for patients to understand and ideally they always carry the card with them.”

The study doctor explains the relevance and indication of patient-initiated **emergency antibiotic therapy**. He describes the different types of stand-by antibiotics based on the emergency card and suggests penicillin as the drug of choice (important: ask whether the patient has a serious penicillin allergy).

Furthermore, the study doctors refers to the medical emergency card and suggests recording severe allergies to antibiotics and the stand-by antibiotic of choice into the designated blank spaces.

**Relevance** of the emergency antibiotic:

- „The connection between a delayed start of appropriate antibiotic treatment and the sepsis mortality has been impressively demonstrated in a number of cohort studies [1].
- If an infection is suspected and an early visit to a doctor (within 2 hours) is not possible, asplenic patients should be equipped with a **stand-by antibiotic for patient-initiated emergency treatment**. Requirement for this procedure is an **extensive patient education**.
- Appropriate for early treatment prior to hospital admission (Stand-by antibiotic therapy) is i.e. amoxicillin/clavulanic acid (50-75 mg/kg per kg of body weight per day split in 3 doses) [1]
- „According to Swiss and German guidelines, permanent antibiotic prophylaxis is only recommened for groups at risk (i.e. patients with a history of previous PSS, patients with a weakened immune system such as patients in the early stages after stem cell transplantation or patients with underlying hematologic malignancies or repeated polychemotherapy).”

Inquire about a known **penicillin allergy**

Refer to the emergency medical card


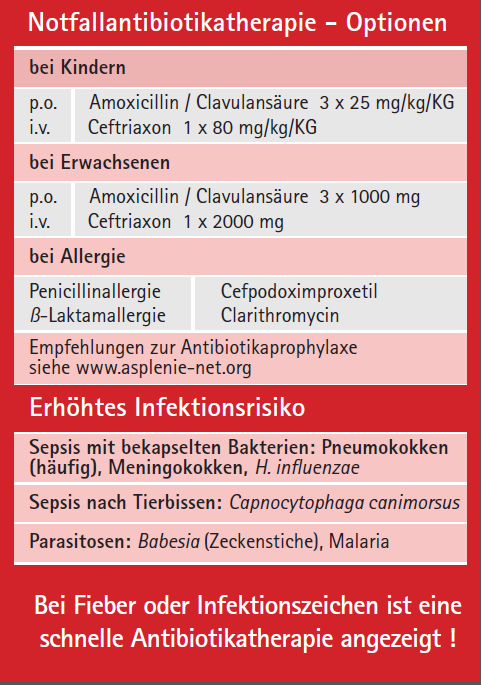


**3.3.3 Conclusion**

The study doctor leaves room for **questions, comments or concerns** about the preventive measures. He also inquires about previously encountered or anticipated **barriers** for the implementation in practice.

He also refers to additional information options and explains the **further proceedings**.

Finally, he **thanks** the physician for participating and says **goodbye**.

„Do you have any **questions or comments** about our call?

„Do you have any **concerns** or anticipate any problems implementing these preventive measures with your patients in practice?

[Or] Have you already encountered difficulties implementing preventive measures into practice?“

„More detailed and updated **information** as well as further links tailored to patients and their physicians can be found on our homepage (www.asplenie-net.org) where you can also order medical emergency cards free of charge.

**Offer to contact** the asplenia outpatient clinic in case of questions or uncertainties

Refer to the further proceedings (next telephone interview and questionnaire to evaluate the training for physicians of the *intervention group*)

**Thank** the physician for the talk and say **goodbye**.

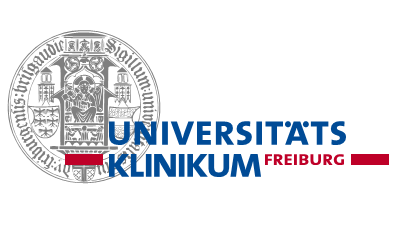
**3.4.1 Information for physicians**

| Information for physicians |
| --- |

**Recommended infection prevention strategies for patients with asplenia: VMA (Vaccinations, Medical emergency card, Antibiotics)**

Asplenic patients have a high lifetime risk for developing a post-splenectomy sepsis (PSS). Compared to the general population the risk of a sepsis-associatiated hospitalisation is increased by 6-fold [1].

1. **Vaccinations**

The Standing Committee on Vaccination (STIKO) of the Robert Koch Institute and the Swiss Federal Office Public Health (FOPH) recommend vaccinations against **pneumococci, meningococci and *Haemophilus influenzae***. Furthermore, the **influenza vaccine** should be administered annually.

- Recommended vaccinations and a vaccination schedule can be found on the medical emergency card, *which is included in this material.*

*On page 3 of this information material, you will also find an overview of the vaccination schedule.*

- Only 6 % of patients being treated in routine care are fully vaccinated according to current guidelines as shown in a study [2].
- It is important that doctors regularly assess a patient’s need for (booster) vaccinations and set up an individualised vaccination schedule.
- Vaccination recommendations are continually updated can be read up on www.asplenie-net.org.

1. **Medical emergency card**

The German Society of Infectious Diseases (DGI) and German Sepsis Society (DSG) in cooperation with further medical societies developed a medical emergency card for patients with asplenia. Patients are advised to carry this card at all times **to inform medical providers about the increased risk of sepsis in case of emergency**.

Moreover, all recommended measures for infection prevention are summarised on the emergency card.

- *A medical emergency card is included within these documents.*
- More copies for distribution to asplenic patients can be ordered via www.asplenie-net.org.
- It is important that doctors educate patients about the purpose of the emergency card as well as filling it out with the patient.

1. **Emergency therapy with a Stand-by Antibiotic**

If infection is suspected, an urgent visit to the primary care provider or closest emergency room is required to initiate antibiotic therapy without delay should sepsis be diagnosed (please refer to the Surviving Sepsis Campaign guidelines [3]).

In case a doctor’s office or emergency room is not immediately accessible, an **early antibiotic therapy can be ensured by patient-initiated emergency therapy when experiencing first symptoms of severe infection**.

- A study showed that only 47 % of asplenic patients received a stand-by antibiotic [2]. Doctors should prescribe an emergency antibiotic and check-in with the patient whether a renewal is needed upon expiration of the old prescription.
- Further research revealed that the majority of patients are insufficiently educated about the life threatening risk of infection and appropriate infection prevention measures [4] leading to a significant increase in risk of PSS compared to patients receiving education (16.4 % versus 1.4 % in educated patients).
- Comprehensive patient education is crucial for a patient controlled emergency therapy and all additional preventive measures.

Detailed information and further links can be found here: **www.asplenie.net.org**

For further questions, please contact the asplenia outpatient clinic at the University Medical Centre Freiburg.

**Literature**

1. Theilacker, C., Ludewig, K., Serr, A., Schimpf, J., Held, J., Bogelein, M., et al. (2016). Overwhelming Postsplenectomy Infection: A Prospective Multicenter Cohort Study. *Clin Infect Dis 1*; *62*(7), 871-878.

2 Hromek, J. (2015). Dissertationsarbeit: Prospektive Kohortenstudie zu Infektions-komplikationen und Prophylaxemaßnahmen bei Patienten nach Splenektomie
http://d-nb.info/1082093920/04

3 Leitlinie der Surviving Sepsis Campaig 2012: http://www.survivingsepsis.org/Guidelines/Pages/default.aspx

4 Hegarty, P. K., Tan, B., O'Sullivan, R., Cronin, C.C., & Brady, M.P. (2000). Prevention of postsplenectomy sepsis: how much do patients know?. *The Hematology Journal*, *1*(5), 357-359.

5 Sayed, M.H (2004). Overwhelming postsplenectomy infection: is quality of patient knowledge enough for prevention? *The Hematology Journal*, *5*(1), 77-80.

**Vaccination schedule for asplenic patients**

**Vaccination schedule Status 25.02.2019**

**Cave: Current recommendations must be verified before using this scheme.**

|  | **Basic immunization** | | **Booster vaccines** |
| --- | --- | --- | --- |
|  | 1. **Vaccination** | 1. **Vaccination** |  |
| **Pneumococci***  Ideally sequential vaccination **first** with Prevenar-13® (PCV-13), **then** Pneumovax® (PSV-23) | Vaccination  (PCV-13) | 2 months later  (PSV-23) | 5-6 years later  (PSV-23) |
| If Pneumovax® (PSV-23) was received first: |  | At earliest 12 months after PSV-23:  (PCV-13) | 5-6 years after last PSV-23:  (PSV-23) |
| **Meningococci***  Nimenrix® **or** Menveo® (Men-ACWY);  Bexsero® **or** Trumenba® (Men-B) | Vaccination  (Men-ACWY) | 2 months later  (Men-ACWY) |  |
|  | Vaccination  (Men-B) | 2 months later  (Men-B) | 4 months later  (only necessary with Trumenba®) |
| ***Haemophilus influenzae* type b**  ACT-HiB® | Vaccination  (HiB conjugate vaccine) | None | - |
| **Flu (Influenza)**  tetravalent | Influenza vaccine | - | Annual |

Status 03/2019

Please document the vaccination dates in the patient’s medical emergency card.

*** Pneumococci, Meningococci:** There are two different vaccination preparations used.

| 4 Literature |
| --- |

1. Theilacker C, Ludewig K, Serr A, Schimpf J, Held J, Bogelein M, et al. Overwhelming Postsplenectomy Infection: A Prospective Multicenter Cohort Study. Clin Infect Dis 2016 Apr 1;62(7):871-878.
2. Edgren G, Almqvist R, Hartman M, Utter GH. Splenectomy and the risk of sepsis: a population-based cohort study. Ann Surg 2014 Dec;260(6):1081-1087.
3. Sumaraju V, Smith LG, Smith SM. Infectious complications in asplenic hosts. Infect Dis Clin North Am 2001 Jun;15(2):551-65, x.

4 Di Sabatino, Carsetti R, Corazza GR. Post-splenectomy and hyposplenic states. Lancet 2011 Jul 2;378(9785):86-97.

5. Hromek J. Dissertationsarbeit: Prospektive Kohortenstudie zu Infektionskomplikationen und Prophylaxemaßnahmen bei Patienten nach Splenektomie (2015); <http://d-nb.info/1082093920/04>

6 Schwarzer R, Schuz B, Ziegelmann JP, Lippke S, Luszczynska A, Scholz U. Adoption and maintenance of four health behaviors: theory-guided longitudinal studies on dental flossing, seat belt use, dietary behavior, and physical activity. Ann Behav Med 2007 Apr;33(2):156-166.

7. Faller, H., Reusch, A. & Meng, K. (2011). Innovative Schulungskonzepte in der medizinischen Rehabilitation. Bundesgesundheitsbl, 54, 444-450.

8. Abraham C, Michie S. A taxonomy of behavior change techniques used ininterventions. Health Psychol 2008 May;27(3):379-387.
