# Supplementary material for: Efficacy of a novel patient-focused intervention aimed at increasing adherence to guideline-based preventive measures in asplenic patients: the PrePSS trial
Source: Infection. 2023 Sep 1;51(6):1787–95. doi: 10.1007/s15010-023-02088-7 (PMC10665246; doi:10.1007/s15010-023-02088-7)

Table S1: Operationalization of the PrePSS-score parameters after weighting of the parameters

**Parameter**	**Score**
**(1) Guideline-conform sequential pneumococcal vaccination**	**Range 0-3**
▪ PCV-13 and PSV-23, correct sequence and intervals[1]	3
▪ PCV-13 and PSV-23, altered sequence or intervals	2
▪ Only PCV-13 or PSV-23	1
▪ No pneumococcal vaccination	0
**(2) Guideline-conform meningococcal vaccination**	**Range 0-3**
▪ Men-ACWY and Men-B complete primary immunization[2]	3
▪ Men-ACWY and Men-B incomplete primary immunization	2
▪ Men-ACWY or Men-B	1
▪ No meningococcal vaccination	0
**(3) Handing-over and carrying a medical alert card**	**Range 0-2**
▪ Medical alert card handed out, permanently carried along (e.g. in purse)	2
▪ Medical alert card handed out but not permanently carried along	1
▪ No medical alert card handed out	0
**(4) Stand by-antibiotic prescribed and available ('pill in the pocket')**	**Range 0-2**
▪ Stand by-antibiotic prescribed, antibiotic permanently available (24 h/7 d)	2
▪ Stand by-antibiotic prescribed, antibiotic not permanently available	1
▪ No stand by-antibiotic prescribed	0
**Total PrePSS-score [Range]**	**0-10**

[1] 13-valent conjugate vaccine PCV-13 (Prevenar-13®) after ≥2 months followed by 23-valent polysaccharide vaccine PSV-23 (Pneumovax®)

[2] Tetravalent meningococcal conjugate vaccination Men-ACWY (Menveo®, Nimenrix®), two doses at least two months apart; meningococcal serotype B vaccine Men-B (Bexsero® [two doses] or Trumenba® [three doses]).

Figure S1: PrePSS-score stratified by the intensity of the physician-centered intervention

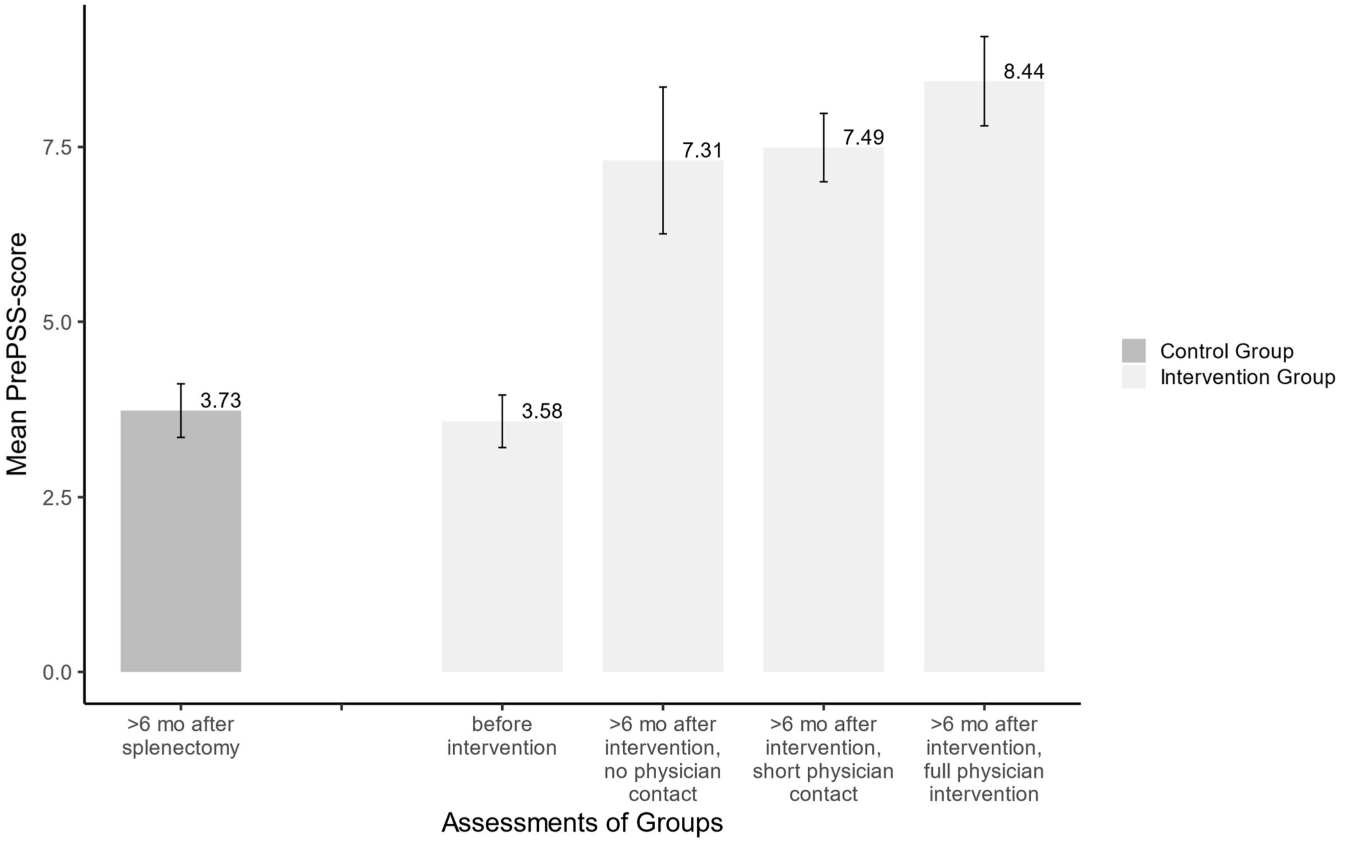

Supplement: Supplementary file 3 — Supplementary file3 (PDF 641 KB) [file 15010_2023_2088_MOESM3_ESM.pdf]
